# Mechanisms of Transfer RNA Fragments Functionality Within and Between Cells and Organisms

**DOI:** 10.3390/cells14211696

**Published:** 2025-10-29

**Authors:** Sathyanarayanan Vaidhyanathan, Yan X. Lin, Adesupo A. Adetowubo, Fatmanur Kiliç, Sai Anusha Jonnalagadda, Andrey Grigoriev

**Affiliations:** 1 Department of Biology, Rutgers University, Camden, NJ 08102, USA; sv646@scarletmail.rutgers.edu (S.V.); aaa586@scarletmail.rutgers.edu (A.A.A.); fk274@scarletmail.rutgers.edu (F.K.); sj1110@scarletmail.rutgers.edu (S.A.J.); 2 Center for Computational and Integrative Biology, Rutgers University, Camden, NJ 08102, USA; yxl1@scarletmail.rutgers.edu

**Keywords:** transfer RNA, transfer RNA fragments, tRF mechanisms, regulation, extracellular vesicles, cell proliferation, cell cycle, inflammation, immune response, biomarkers

## Abstract

Transfer RNA-derived fragments (tRFs) have become a significant category of small non-coding RNAs that likely play vital roles in various cellular functions. Initially, research on small RNAs overlooked tRFs as simple byproducts of tRNA degradation, but recent findings show they are precisely produced molecules that regulate gene expression. Studies have demonstrated that tRFs regulate genes and proteins through various mechanisms, from miRNA-like targeting that relies on Argonaute (AGO) protein to lesser-known modes of action. Recent reports also suggest that tRFs are involved in multiple diseases, including cancer, where they may be utilized as biomarkers. Notably, tRFs can be transported between different cells and tissues of an organism or even across different organisms, further emphasizing their biological significance. Although evidence increasingly indicates that tRFs may function as new regulatory agents in health and disease, their biogenesis and underlying mechanisms are not yet fully understood. Conducting a thorough exploratory analysis of the tRF modes of action could be a valuable resource for advancing this growing field. Our goal in this review is to gather and examine the latest research on tRF biology, focusing on its diverse and dynamic molecular mechanisms discovered in different disease contexts, with a view toward potential applications in medicine. We aim to gain a deeper understanding of tRFs and explore their potential for new therapeutic breakthroughs by combining insights from molecular studies, disease models, and clinical research.

## 1. Introduction

Transfer RNA-derived fragments (tRFs) have emerged as a versatile class of small RNA (sRNA) with diverse regulatory functions in cellular physiology and pathology. Initially regarded as degradation byproducts, tRFs are now recognized as precisely generated molecules that modulate gene expression through mechanisms including Argonaute (AGO)-dependent silencing, mRNA stability control, ribosome targeting, direct protein binding, and structural modulation [1,2,3,4,5,6,7,8]. These activities place tRFs at the intersection of transcriptional, translational, and post-transcriptional regulation, influencing cell proliferation, apoptosis, inflammation, stress adaptation, and differentiation [9,10,11,12,13].

Beyond their intracellular roles, tRFs can be packaged into extracellular vesicles such as exosomes, enabling long-range intercellular communication [14,15]. Exosomal tRFs have been implicated in neural development and neuroprotection, cardiovascular remodeling, immune modulation, and tissue regeneration [16,17,18,19]. Distinct tRF abundance profiles have been documented in cancers, cardiovascular diseases, and inflammatory conditions, underscoring their potential as diagnostic biomarkers and therapeutic targets [7,11,20,21,22].

tRFs are also known to be generated under certain conditions, like cellular stress; however, they have also been found in non-stress conditions [23]. The nature of tRFs in regulating potentially vital cellular functions and biological processes in organisms renders them a distinctive molecule worthy of exploration, despite the limited understanding of their biogenesis and roles. We do not cover details of tRF biogenesis here and instead refer to a comprehensive tRF-focused review from the lab that first described these molecules [24] and a recent update from a broader sRNA biogenesis review [25].

This is our second paper in a series of reviews highlighting different aspects of tRFs. In the first paper (we call it DISEASE REVIEW here), we went over a large number of studies of tRF in multiple diseases, including cancer and diseases of the nervous, cardiovascular, and musculoskeletal systems [26]. Comparing ~100 studies, we identified three different types of papers based on the depth of the evidence provided. Such evidence ranged from RT-qPCR measurements of tRF levels and purely computational prediction of their possible targets (Type I) to confirmation of target binding, e.g., with luciferase assays (Type II); to additional disease-specific validation, in vitro and in vivo testing, often involving transfection of tRF mimics or antisense oligos; to other experiments that provide further compelling evidence to elucidate the roles of tRFs (Type III). Here, whenever possible, we focus mainly on Type III studies, as they offer the most reliable information.

The current review synthesizes current knowledge of tRF mechanistic diversity, extracellular transport, and functional roles in cellular and immune regulation. By integrating evidence from molecular studies, disease models, and clinical observations, we provide a comprehensive perspective on how tRFs influence cell proliferation, cell cycle progression, inflammatory pathways, and immune signaling. We further discuss their relevance to disease and highlight how their unique properties may be harnessed for translational applications in precision medicine.

### Methodological Advances, Experimental and Computational

Complementary DNA (cDNA) libraries for sRNA sequencing (sRNA-seq) are produced via adapter ligation to the 3′ and 5′ ends of sRNAs, followed by reverse transcription, an efficient protocol for sRNA species with a 5′ phosphate (5′-P) and 3′ hydroxyl (3′-OH). This approach works well for miRNAs but not for sRNAs with modifications. Problematic modifications include 3′-phosphate (3′-P) and 2′,3′-cyclic phosphate (2′3′-cP), which block the adapter ligation [27], and RNA methylation varieties that interfere with reverse transcription [28,29]. sRNA molecules with such modifications (tRNAs and tRFs are the prime examples, as they carry a multitude of modifications) are often poorly converted into cDNAs, resulting in problematic detection/quantitation by sRNA-seq.

To address these issues, enzymatic treatment protocols have been developed to demethylate methylated RNA bases for enabling reverse transcription [28,29], and to convert the 3′-P or 2′3′-cP into 3′-OH and to add a 5′-terminal phosphate (5′-P), facilitating adapter ligation for sRNA-seq [30]. In addition to such methods, specific to certain types of modification, approaches addressing broader sets of modifications, such as PANDORA-seq (panoramic RNA display by overcoming RNA modification aborted sequencing), have been published [31].

Identification of tRF targets and target-binding regions remains one of the main challenges, so other methods have been developed to address such challenges. For instance, crosslinking, ligation, and immunoprecipitation (CLIP-Seq) is used to identify crosslinking sites between sRNA and proteins. The process involves UV irradiation to crosslink RNA with RNA-binding proteins, forming covalent bonds. The resulting complex is immunoprecipitated, and its cDNA is sequenced [32]. While this method identifies binding sites on the sRNA, it does not detect modifications present in the sRNA or its targets.

A modification of CLIP-Seq, CLEAR (Covalent Ligation of Endogenous Argonaute-bound RNAs)-CLIP, allows one to detect targets by stabilizing the connections between RNA and RNA-binding proteins, such as AGO, and between the sRNA and its target. This procedure incorporates crosslinking, ligation of the sRNA and target ends, and subsequent immunoprecipitation of the complex, followed by sequencing [33]. This method is similar to Crosslinking, Ligation, and Sequencing of Hybrids (CLASH), which also captures sRNA loaded into AGO proteins along with their targets by ligating them together and then immunoprecipitating. Their cDNA sequences reveal *chimeric reads*, each containing both sRNA and its putative target [34]. Originally developed to investigate miRNA targets, CLASH and CLEAR-CLIP were also used to study other sRNAs like tRFs. Although both methods can identify potential sRNA–target pairs, these pairs require validation through further experiments such as RT-qPCR or luciferase assays.

A method with a rather complex name, T4 Rnl2/AP/NaIO4/T4 Pnk (3′ phosphatase minus)/RtcB-based sRNA-seq (TANT-seq) simultaneously captures modifications (3′-OH or 3′-cP) of the sRNAs and enables the identification of low-abundance sRNAs, 15 to 30 nucleotides (nt) in length, with modifications at their 3′-end, all with high sensitivity. To validate the abundance of different types of modified sRNAs, the TE-qPCR technique is used to quantify their levels via qPCR [35]. Furthermore, additional experiments, such as those using mimics of the sRNAs, are necessary to validate the target sets of the sRNAs predicted by computational tools, as TANT-Seq does not detect them.

Despite the development of such powerful methods, most of the disease studies we reviewed follow the standard cDNA route for initial assessment of tRF abundance, later validated by RT-qPCR. Thus, our strong preference for type III papers ensures that their results are trustworthy, and the mechanisms of action of tRFs described are supported by multiple lines of evidence. As we enumerate various experiments, each possessing its own limitations and advantages, it is evident that integrating results from different experimental methods, along with computational tools, enables a more complete understanding of sRNA function. For instance, combining experiments like ribosome profiling (Ribo-Seq), which capture snapshots of active ribosomes in a cell at specific times, with RNA-seq or other relevant sequence data can provide essential insights into the roles of sRNAs and their effects on gene regulation [36]. This combined approach also helps clarify their potential roles as gene expression regulators, offering a deeper understanding of their mechanisms and functions. Ideally, expanding our knowledge in this area could significantly improve therapeutic strategies and treatments.

tRFs have also been identified in other organisms, including plants [37], flies [38,39], mice [40] and rats [41], bacteria [42], and viruses [43,44]. While these facts point to evolutionary conservation across kingdoms of life, we are not aware of specific studies aiming to confirm such conservation. However, in our own work on *Drosophila* [39] and rat brain [41], we observed conservation of potential tRF binding sites, determined computationally, among putative animal target gene homologs. Upon our re-analysis of CLASH results, we could identify common motifs between different targets of the same tRF inferred from chimeric reads [45,46]. Analyzing conservation of 7-mers among 100 animal genomes within such motifs, we described peaks of such conservation near the starts of such motifs [45].

We made the results of these large-scale computational analyses and potentially interacting tRF–target pairs available as an online database, tatDB [47], and there exist other similar databases. The tRFTar [48] database contains tRF–target pairs from 146 CLIP-seq datasets, with co-expression profiles of the tRFs and their targets, thus assisting in exploring the role of tRFs. tsRFun [49] utilizes various sequencing datasets from different experimental methods to evaluate tRF’s prognostic significance across 32 cancers, with interaction networks involving tRFs, miRNAs, and mRNAs. tRFtarget [50] compiles information about tRFs and their targets from databases above and from predictions of its tRF–target pipeline across nine different species. However, stringent additional experimental validation of these (or novel) pairs is important for establishing the functionality of tRFs.

There are also growing efforts in predicting tRF targets by machine learning methods. One of the first such predictors for tRFs, tRForest [51], uses Random Forest and considers only CLASH data for 3′UTRs as target regions, excluding coding sequences, 5′UTRs, and introns, although the authors acknowledge these are also targeted. HybriDetector [52] can identify guide–target pairs from chimeric reads produced by sequencing experiments such as CLASH, CLEAR-CLIP, and miR-eCLIP. It works with tRFs, although the focus in the paper was primarily on miRNAs.

Furthermore, there are many other binding sites or hybridization prediction tools like TargetScan [53], RNAhybrid [54], and miRanda [55], which are designed primarily to identify miRNA targets as they target genes, with a seed region located at positions 2–7 nt within them. These tools were often used in the publications we reviewed. However, tRFs do not necessarily bind to targets through such miRNA-like seed regions, so the predictions by these tools should be interpreted carefully when used with tRFs and their targets, as they often fail in validation [7,56]. However, the development of tRF-specific tools could help to advance the field and broaden our understanding of this domain by reducing extensive experimental efforts and facilitating progress in research.

In the DISEASE REVIEW, we also had to deal with >100 different tRFs as we focused on the breadth of studies involving them. Since the original reviewed publications utilized various naming schemes, we described those schemes in detail and tried to unify tRFs at the level of the sequence [26]. As a side result, this allowed us to detect identical tRFs originally described in divergent disease contexts, and we have published a comparative study of such events [57], where the naming scheme of the tatDB database was used [47]. In the current review, we do not concentrate on tRF sequences but on the description of mechanisms reported for them, and on the aspects of cellular and organismal processes involving tRFs. Hence, we utilize the tRF names from the original publications here, as it could help the readers interested in specific details to find tRFs in those original papers (as sequences are not always easy to find there and they would only present an obstacle to such users).

## 2. Mechanisms of tRF Action

**Figure 1:**
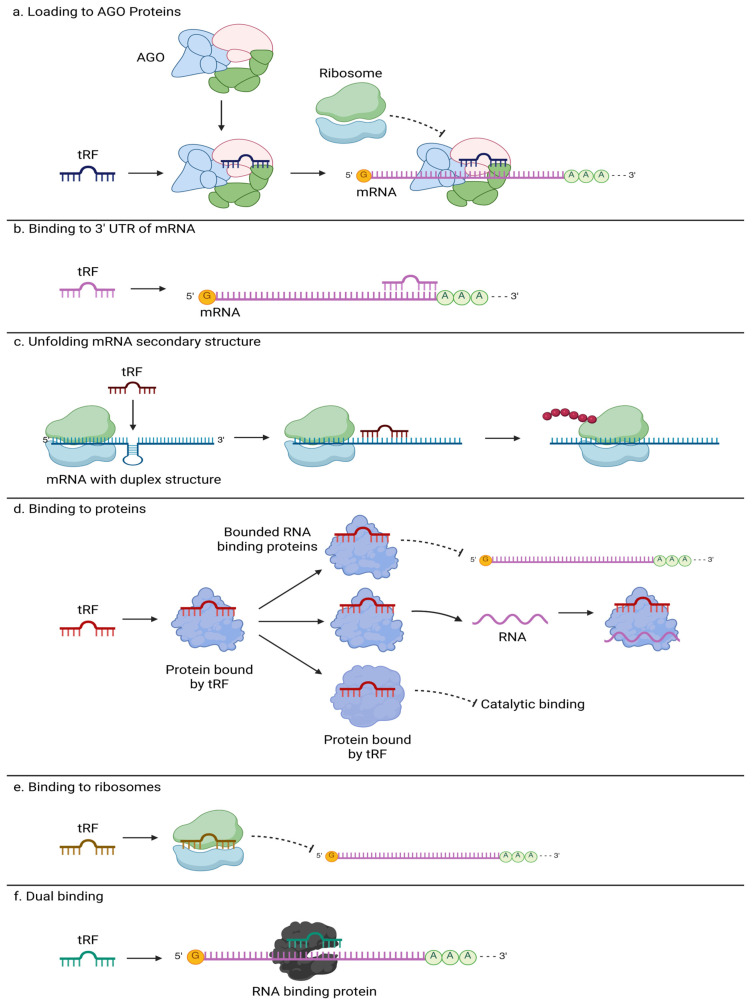
Illustrated mechanisms of action for tRNA-derived fragments (tRFs). (**a**) **Loading to AGO proteins:** tRFs bind to Argonaute proteins to form RNA-induced silencing complexes (RISCs), guiding them to complementary mRNAs for translational repression or degradation. (**b**) **Binding to 3′UTR of mRNA:** Direct association with target mRNA 3′UTRs to alter stability or translation. (**c**) **Unfolding mRNA secondary structures:** Remodeling duplexed mRNA regions to facilitate ribosome access and enhance translation. (**d**) **Binding to proteins:** Interaction with RBPs, potentially altering RNA processing or translation, including modulation via G-quadruplex formation. (**e**) **Binding to ribosomes:** Direct association with ribosomal subunits to modulate translation, in some cases stalling elongation. (**f**) **Dual binding:** Simultaneous interaction with both non-AGO RBPs and target mRNAs, enabling coordinated regulation at multiple levels.

One view of how tRFs interact with their targets is through mechanisms that closely resemble those of microRNA (miRNA), primarily involving Argonaute (AGO) proteins and complementary binding to specific mRNA transcripts [7]. For instance, tRFs bind to AGO proteins, such as AGO1-4 in humans, forming RNA-induced silencing complexes (RISCs) guided to complementary mRNA targets, modulating gene expression by inhibiting translation or destabilizing the transcripts (Figure 1a). Binding to a target is initiated via a short “seed” region of several nt in length (similar to 7–8 nt long miRNA “seeds”). A notable example is a 22-nt tRF designated CU1276, shown to bind AGO proteins and repress *RPA1* expression by targeting its 3′ untranslated region (3′UTR). This repression was found to be seed-sequence-dependent and abolished when the binding site was mutated, confirming a microRNA-like mode of action through AGO-mediated silencing. In some contexts, specific tRFs interact with AGO2 to inhibit transcription factors like *STAT3*, contributing to downstream regulatory changes in cellular signaling. For example, tRF-33-P4R8YP9LON4VDP was shown to directly bind the 3′UTR of *STAT3* and reduce its expression in an AGO2-dependent manner, further blocking the transcription of the downstream genes [58].

Apart from AGO-dependent silencing, tRFs can bind directly to the 3′UTR of target mRNAs, destabilizing the transcripts or altering their stability and translation (Figure 1b). In the case of regulatory gene targets, this may lead to significant downstream changes. For example, 5′tiRNA-Cys-GCA targets the 3′UTR of the *STAT4* gene in vascular smooth muscle cells, reducing mRNA stability and translation, which inhibits cell proliferation and migration [8]. They validated this mechanism through RNA pulldown, luciferase reporter assays, and functional experiments that confirmed the biological impact of *STAT4* repression. Similarly, tRF-Glu-TTC-047 was reported to bind the 3′UTR of *IGF1* mRNA to suppress its expression, influencing pathways involved in cell growth and protein synthesis [2]. In a study of cardiac hypertrophy in cardiomyocytes, Gly-CCC-derived tRF, called tRFs1, was found to bind the 3′UTR of the *Timp3*, cardiac hypertrophy regulatory factor gene, repressing it. Mimic of tRFs1 also increased the expression of hypertrophic markers, such as *ANF*, *BNP*, and *β-MHC* [19], supporting cell growth and hypertrophic development, and it may be related to such repression of *Timp3*. Another tRF-Gly-GCC tRF targets *ICAM1* and *MHC* mRNAs, modulating their stability and translation, which in turn affects cell adhesion and phenotype switching in atherosclerosis [59].

Another reported mechanism of interacting with their targets involves the ability of tRFs to unfold mRNA secondary structures to enhance translation. For instance, LeuCAG 3′tRF binds to the mRNA of ribosomal protein S28 (*RPS28*). This interaction occurs at defined sites within the mRNA, where the tRF-driven unfolding of duplexed regions facilitates access to the translation machinery, thereby promoting efficient protein synthesis (Figure 1c). Notably, when the tRF binding sites are mutated, translation of *RPS28* is significantly reduced [6]. This mechanism illustrates how tRFs can modulate gene expression not only through transcript repression but also by promoting translation.

In addition to transcript-level regulation, recent findings demonstrate that certain tRFs can directly interact with proteins, thereby influencing post-transcriptional processes (Figure 1d). Using RNA pulldown assays coupled with mass spectrometry, the 3′ tRNA half tDR-36:74-Asn-GTT-2-M2, upregulated in Klotho knockout, was found to bind directly to multiple RNA-binding proteins (RBPs) involved in RNA splicing, mRNA decay, and pseudo-uridylation [1]. Noteworthy interactions included spliceosome components such as *HNRNPF*, *SNRNP70*, and *SART3*, as well as members of the SKI complex like *SKIV2L*, suggesting a regulatory role in RNA processing pathways. Moreover, the guanine-rich sequence of tDR-36:74-Asn-GTT-2-M2 suggests its potential to form G-quadruplexes, stable secondary structures characterized by stacked guanine tetrads [1]. It was previously demonstrated that G-quadruplex-forming tRNA fragments can regulate translation initiation and promote stress granule formation, implicating these structures in neuroprotection [5]. This dual capacity, direct protein binding, and structural modulation via quadruplex formation, expands the functional repertoire of tRFs beyond nucleic acid-based targeting, highlighting their versatile roles in RNA metabolism and post-transcriptional regulation. With regard to protein binding, very recent papers exemplified tRF interactions with other types of proteins, such as Toll-like receptors [60,61], which points to tRF signaling between cells, a role that will be more broadly described in the next sections.

A distinct mechanism involves the direct interaction of tRFs with the ribosome itself, independent of mRNA targeting (Figure 1e). A 26-nt 5′Val-tRF from *Haloferax volcanii* was reported to bind directly to the 30S small ribosomal subunit. This association was shown to be stress-responsive and enriched under elevated pH conditions [3]. Importantly, Val-tRF inhibited protein synthesis in vitro by interfering with peptidyl transferase activity, resulting in a ~45% reduction in translation efficiency. The effect was sequence-specific, as scrambled or unrelated tRFs did not elicit the same inhibition. This finding provided one of the first examples of a tRF functioning as a ribosome-targeting regulatory RNA, suggesting that tRFs can influence translation not only by acting on mRNA or protein partners but also by directly modulating ribosome function.

The same lab has found another particularly novel twist on ribosome-associated tRF function in a 35-nt 5′tRNA-Pro half that binds directly to the 80S ribosome in mammalian cells. Unlike previously described tRFs that inhibit translation initiation, this tRNA-Pro half leads to ribosome stalling and the accumulation of a distinct translational byproduct named ProTiP, identified as a peptidyl-tRNA [4]. ProTiP formation is translation-dependent and occurs both in vitro and in vivo, suggesting that the tRNA-Pro half interferes with translation elongation, rather than initiation. The tRF crosslinks to 18S rRNA at the subunit interface, and its effects are not reproduced by DNA analogs, highlighting a unique, structured RNA-mediated mechanism [4]. This study expands the scope of tRF–ribosome interactions by revealing a stalling mechanism that promotes the accumulation of peptide-tRNA hybrids, further underscoring the functional diversity of ribosome-targeting tRFs.

Another tRF-mediated mechanism involving a dual interaction with a non-AGO protein and mRNA was recently reported (Figure 1f). 5′LysTTT tRF was shown to simultaneously bind the RBP HNRNPM and the 3′UTR of *CHAC1* mRNA. Binding to HNRNPM influences cellular RNA processing and splicing dynamics. Concurrently, direct interaction of 5′LysTTT with the *CHAC1* mRNA’s 3′UTR suppresses *CHAC1* expression, reducing ferroptosis and promoting neuronal survival under stress induced by botulinum neurotoxin [62]. This dual interaction mechanism underscores the multifunctionality of tRFs, as they coordinate post-transcriptional regulation by integrating direct transcript modulation with protein-level interference to maintain neuronal viability.

## 3. Transport of tRFs in Extracellular Vesicles

As implied by the mechanisms described above, tRFs are always close to mRNAs and/or ribosomes. Such closeness was proposed as a factor that might have led to the emergence of primordial RNA interference mechanisms from randomly broken tRNAs [63]. However, cases of transporting tRFs to other cells (or even organisms, as in host-pathogen interactions) are increasingly being reported. Below, we review some illustrative examples, based on loading tRF into extracellular vesicles (EVs) or exosomes (we will use these terms interchangeably here).

**Figure 2:**
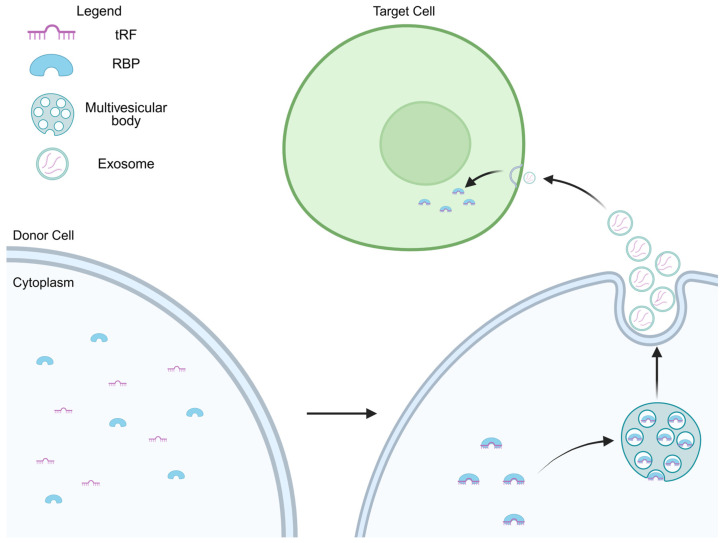
Schematic representation of exosome-mediated transfer of tRNA-derived fragments (tRFs). The figure illustrates how a donor cell (bottom) packages and delivers molecular cargo (tRFs) to a target cell (top) via exosomes. The process begins in the cytoplasm of the donor cell, where tRFs and RBPs are located. This cargo is enclosed within vesicles inside a multivesicular body (MVB). Black arrows show the pathway direction. The MVB then fuses with the donor cell’s plasma membrane, releasing the vesicles into the extracellular space. These vesicles are now called exosomes. The exosomes, containing the tRFs and RBPs, travel toward and are taken up by a nearby target cell. Inside the target cell, the exosomes release their contents into the cytoplasm, delivering the molecular cargo from the donor cell, which can influence cellular processes such as gene regulation, signaling, and homeostasis.

Exosomes are small membrane-bound vesicles (30–100 nm in diameter) secreted by a variety of cell types and found in biological fluids such as plasma, urine, and saliva. They carry specific molecular cargo (including lipids, proteins, and RNAs) reflecting physiological or pathological states of their cells of origin (Figure 2). In this context, RNA-mediated signaling is often considered through the lens of miRNAs that regulate gene expression and long non-coding RNAs that influence the recipient cells’ behavior, for instance, cardiomyocyte proliferation and survival [64,65]. The presence and abundance patterns of exosomal tRFs are beginning to be explored, offering new insights into their potential role in disease detection and cell-to-cell signaling [66].

Notably, tRFs can also be transferred via EVs to recipient cells in a cardiovascular context, modulating protein translation, proliferation, and stress responses via conserved mechanisms of RNA-based intercellular communication [67]. Exosomal tRF-1003 was identified as a regulator in endothelial cell angiogenesis, enhancing it in vitro and in vivo by activating the HIF-1α/VEGF signaling pathway through the downregulation of *MAPK1* expression [68]. In isoproterenol-induced hypertrophy models, altered levels of tRFs1 and tRFs2 increased cardiomyocyte surface area and upregulated hypertrophy markers such as *ANF*, *BNP*, and *β-MHC*, with luciferase assays confirming *Timp3* downregulation [19]. In maternal serum samples, tRF-58:74-Gly-GCC-1 and tiRNA-1:35-Leu-CAG-1-M2 displayed opposing abundance patterns, supporting their role in developmental signaling and cardiac morphogenesis [69].

Exosomes derived from mesenchymal stem cells (MSCs) were shown to protect retinal photoreceptors and modulate immune pathways, associating upregulation of two tRFs with reduced microglial activation and inflammation [16]. Exosomes transported molecules such as tRF-41, which modulated cellular processes in the nervous system [9]; this mechanism is discussed in greater detail in Section 5.

Exosomes also contribute to skeletal and immune homeostasis. MSC-derived exosomes regulate macrophage polarization, suppressing M1-type pro-inflammatory markers (*CD80*, *NOS2*, *MCP-1*) and enhancing M2 markers (*CD206*, *ARG1*, *MRC2*). tsRNA-21109 was upregulated in macrophages treated with MSC-derived exosomes and was negatively correlated with inflammatory activity, underscoring its importance in immune homeostasis [17]. Another study showed that T cell activation induced the EV-mediated release of a specific set of tRFs, while inhibition of EV biogenesis pathways led to the accumulation of these activation-induced EV-enriched tRFs within multivesicular bodies [15] (Figure 2). In bone metabolism, elevated exosomal tRF-25, tRF-38, and tRF-18 were associated with PI3K-AKT and Wnt pathways that influenced osteoblast and osteoclast activity [70].

Within tissue microenvironments, exosomes act as critical mediators of intercellular communication. EVs carried integrins (ITGs) that dictated organ-targeting behaviors by engaging with specific stromal cells to establish pre-metastatic niches. For instance, EVs with ITGαvβ5 preferentially interacted with Kupffer cells in the liver, whereas ITGα6β4 and ITGα6β1 facilitated targeting of lung fibroblasts and epithelial cells [14]. These interactions fostered microenvironments supportive of cellular migration and adhesion. In addition, exosomes stimulated interferon-stimulated gene activation in certain cell types through stromal interactions [71,72]. Stromal-derived exosomal RNA activated *RIG-I* in recipient cells, driving *STAT1*-mediated transcription and amplifying *NOTCH3* responses, thereby reprogramming recipient cells and influencing their plasticity and differentiation [73].

In addition to intercellular communication, tRFs play essential roles between organisms and across different kingdoms of life, influencing interactions among various species via exosomes. For example, tRFs found in outer membrane vesicles (OMVs) of bacteria have been shown to reduce the infectivity of bacteriophages upon encountering them. When the P22 bacteriophage infects *Salmonella*, the tRFs in OMVs help inhibit this infection and protect the bacteria from attack by the bacteriophage. Specifically, tRNA-Thr-CGT-1-1, 44–73 (30 nt), is fully complementary to 29 annotated *Salmonella enterica*-infecting bacteriophages, including P22. This tRF found in OMVs plays a role in decreasing the infectivity of P22. Meanwhile, lower levels of tRFs in secreted OMVs increase infection in a dose-dependent manner, indicating that tRFs are involved in the bacteria’s innate antiviral defense [42].

In humans, tRFs found in saliva can affect host-oral microbial interactions. Host-derived tsRNAs, such as tsRNA-000794 and tsRNA-020498, exhibit high sequence similarity to tsRNAs found in gram-negative oral bacteria, including *Fusobacterium nucleatum*. Upon exposure to *F. nucleatum* by normal human oral keratinocyte-spontaneously immortalized (NOKSI) cells, these cells release specific tsRNAs via exosomes, which serve to inhibit bacterial proliferation by hindering bacterial protein synthesis. These two tRFs were also found not to inhibit the growth of *Streptococcus mitis,* a health-associated gram-positive bacterium, showing the specific nature of action [74]. Another study substituted the 2′-hydroxyl groups with 2′-methoxy groups at the 5′ and 3′ ends of these tRFs, increasing their stability and enhancing inhibition by a 1000-fold, while still maintaining species specificity. The modified tRFs were shown to inhibit *F. nucleatum* clinical tumor isolates. Mechanistic investigations suggested these tRFs might target ribosomes, stopping protein translation [75]. This suggests that tRFs could serve as a host defense mechanism, potentially inhibiting the growth of gram-negative bacteria and supporting oral health.

Novel tRFs and tRNA halves from non-pathogenic *Escherichia coli* strains (NPECSs) within the gut microbiota have been shown to have anticancer properties. These bacterial-derived tRFs and tRNA halves exhibit significant cytotoxicity against colorectal cancer (CRC) cells, with tRF mimics demonstrating greater efficacy than tRNA halves. A 22-nt 5′-tRF derived from tRNA-Leu-CAA (a mimic) from NPECS demonstrated the highest potency against colorectal cancer cells. Its cytotoxic effects can be further strengthened through chemical modifications, such as 2′-O-methylation of guanosine, which stabilizes its tertiary structure. The modifications were also observed to enhance the inhibitory effects of the mimics on colony formation and migration of CRC cells. This discovery underscores the potential of tRFs as a novel class of bioactive molecules produced by gut microorganisms, with significant implications for the development of innovative cancer therapies [76].

These findings suggest that exosomal tRFs play a crucial role as mediators in various biological interactions across domains of life, ranging from microbial defense against viruses to host-microbe communication, and even influencing host disease conditions.

## 4. Regulation of Cell Proliferation and Cell Cycle

tRFs modulate cell cycle dynamics and survival pathways that govern cellular proliferation. For instance, tRF-Leu-AAG was shown to enhance proliferation by downregulating UPF1, a key player in mRNA surveillance and stability, thereby promoting cell cycle progression and suppressing apoptotic signaling [12]. Similarly, tRF-5026a was found to regulate the PI3K/AKT signaling pathway, which is one of the most conserved and widely activated pathways in cell growth and metabolism. Over-abundance of tRF-5026a suppressed PI3K and AKT protein expression, while simultaneously enhancing the levels of PTEN, a negative regulator of the pathway. This leads to inhibition of cell proliferation, reduced migration, G0/G1 cell cycle arrest, and decreased colony formation. Conversely, depletion of tRF-5026a activated the PI3K/AKT axis and enhanced proliferative potential, confirming that this tRF functions as a regulator in proliferative signaling [13].

Adding to this, tRF-Gln-CTG was found to promote proliferation in vascular smooth muscle cells (VSMCs) following arterial injury. In a rat carotid artery injury model, tRFGlnCTG level increased in VSMCs, where it enhanced both proliferation and migration. Functional experiments showed that synthetic mimics of tRFGlnCTG elevated VSMC viability, while its inhibitors reduced these effects. tRFGlnCTG was found to downregulate FAS, a gene central to apoptotic signaling. Overexpression of FAS reversed the proliferative effects of tRFGlnCTG, indicating that this tRF modulates cell growth by suppressing apoptosis pathways [77].

While many tRFs promote proliferation, others act as negative regulators. tRFs derived from tRNA^Val^ and tRNA^Gly^ are upregulated under ischemic and hypoxic stress conditions. When synthetically introduced into endothelial cells, these tRFs suppressed proliferation, migration, and tube formation, thereby inhibiting angiogenesis and vascular remodeling in response to oxygen deprivation, illustrating how tRFs can function as molecular brakes during stress adaptation [78].

Emerging evidence also highlights the role of mitochondrial-derived tRNA fragments in regulating muscle-specific cell states. mt-Ty 5′tiRNAs, which were shown to be highly enriched in skeletal muscle and heart, increased with developmental age. In C2C12 myoblasts, gapmer-mediated knockdown of mt-Ty 5′tiRNAs impaired both proliferation and differentiation, elevated apoptosis, and caused mitochondrial fragmentation. RNA-Seq and qPCR analyses showed that knockdown led to the downregulation of genes critical for myogenic progression and cell cycle control, such as *Myog, Pax7*, and *Ttn*, and disrupted stress-responsive pathways including PI3K-Akt, FoxO, and p53. Conversely, an increase in mt-Ty 5′tiRNAs partially restored the expression of these genes and upregulated others like *Egr1* and *Prl2c2*, supporting their role as positive regulators of skeletal muscle development [10].

In a murine model of aortic dissection (AD), systemic administration of 5′-tiRNA-Cys-GCA via tail vein injection significantly reversed disease progression and improved survival outcomes. This tRF was found to be markedly downregulated in both human AD tissues and AD model mice, particularly in VSMCs. Its elevation by means of the added tRF mimic suppressed VSMC proliferation, migration, and phenotypic switching, while knockdown had the opposite effect. 5′-tiRNA-Cys-GCA was reported to act by directly binding and downregulating *STAT4*, a transcription factor involved in vascular remodeling. These findings highlight a protective role for this tRF in maintaining VSMC contractile phenotype and suppressing pathological remodeling during aortic dissection, suggesting that specific tRFs can modulate disease-relevant gene expression programs in a cell-type-specific manner [8].

## 5. Inflammatory Regulation and Immune Signaling

tRF levels are sensitive to inflammatory stimuli, and several tRFs were identified as modulators of immune signaling. Among them, tRF-21 was repressed by proinflammatory cytokines such as IL-6 and LIF. These cytokines downregulated the RNA-binding protein SRSF5, which was shown to interact with tRNA^GlyGCC^ and facilitate the generation of tRF-21. Reduced SRSF5 levels therefore suppressed tRF-21 biogenesis. Loss of tRF-21 enabled increased phosphorylation of hnRNPL by AKT2/1 and enhanced the formation of an hnRNPL–DDX17 splicing complex, which drove the alternative splicing of transcripts such as *Caspase* 9 and *mH2A1*, promoting cell survival and migration. Under normal conditions, tRF-21 was shown to help limit inflammation-induced invasive responses [11].

Other tRFs also modulate inflammatory pathways. tRF-41, for example, was highly enriched in exosomes released under inflammatory conditions. Upon transferring to recipient cells, it enhanced the expression of cytokines such as IL-1β and IL-6, while suppressing growth-related genes like *IGF-1* and *Wnt3a*. The inflammatory activity of tRF-41 was partially mediated through the inhibition of Wnt/β-catenin signaling, resulting in elevated apoptosis and altered cell cycle progression. These findings suggest a role for tRFs not only in initiating inflammation but also in regulating the resolution and repair phases [9].

A study investigating myocardial ischemia in rats found that caloric restriction (CR) modulates tRF levels, contributing to cardio protection partly through inflammatory signaling pathways. Specifically, CR pretreatment reduced levels of several tRFs that were dysregulated during myocardial ischemia, including tRF-Gly-TCC-018, tRF-Cys-GCA-022, and tRF-Met-CAT-008. These tRFs were shown to target mRNAs involved in macromolecular metabolism and apoptosis, such as *Casp2* and *Rbfox1*, with functional experiments confirming that altering tRF levels led to corresponding changes in target gene expression. Notably, many of the regulated target genes were linked to oxidative stress, metabolic dysfunction, and inflammatory cascades that contributed to tissue injury. These findings highlight the potential of tRFs to act as mediators of inflammation and stress adaptation in ischemic tissue [22].

Transcriptomic studies in Huntington’s disease (HD) models revealed an enrichment of specific 5′ tRNA-derived fragments in inflamed brain regions, highlighting their potential role in regulating immune responses. Among these, 5′ tRF-Ala emerged as both neurotoxic in vitro and a likely contributor to inflammatory signaling. Although 5′ tRF-Gly and 5′ tRF-Val did not display direct neurotoxicity in neuronal assays, they were markedly elevated in HD putamen. Notably, administration of HD-derived sRNAs that encompassed these tRFs induced robust expression of proinflammatory cytokines, including IL-1β, CXCL2, PTGS2, and TNF, along with a transcriptional shift favoring glial activation signatures. These findings support a model in which tRFs (particularly 5′ tRF-Ala) serve as upstream regulators of neuroinflammatory pathways in the diseased brain [79].

## 6. Conclusions

tRFs have emerged as a rapidly growing frontier in RNA biology, linking fundamental molecular mechanisms with wide-ranging physiological effects. They regulate gene expression through multiple pathways, including AGO-dependent repression, mRNA destabilization, translation modulation, protein interactions, and ribosome targeting, demonstrating a versatility comparable to that of canonical sRNAs such as miRNAs [1,2,3,4,5,6,7,8]. Moreover, exosome-mediated tRF transfer adds another layer of regulatory complexity, enabling communication across tissues and even organisms with implications for development, immune responses, tissue repair, and disease progression [11,16,17].

tRFs exhibit distinct abundance patterns and mechanistic roles in many conditions, including cancer, cardiovascular disorders, and inflammatory pathologies, underscoring their potential as diagnostic and therapeutic targets [8,11,20,22]. The growing body of high-evidence studies confirms that tRFs are not mere degradation products but active participants in cellular adaptation and pathology [7,58]. Future research should focus on elucidating their biogenesis, context-specific targeting rules, and in vivo functional networks, while advancing strategies for clinical translation, including biomarker development and tRF-based therapeutics [11].

By bridging molecular biology, systems physiology, and translational research, tRF investigations are poised to redefine our understanding of RNA-mediated regulation and to open new avenues for targeted intervention in human disease [7,11].

## Data Availability

Not applicable.
